# Building the foundation: Training, stakeholder engagement, and planning for Common Elements Treatment Approach (CETA) implementation in Uganda

**DOI:** 10.1371/journal.pgph.0006566

**Published:** 2026-06-12

**Authors:** Amanda P. Miller, Carlos D. Rivera Saldaña, Susan M. Kiene, Emmanuel Menya, Caleb Figge, Hafsa Lukwata Sentongo, William Byansi, Stephen Mugamba, Kenneth Kalani Okware, Doreen Tuhebwe, Laura Murray, David Mwanza, Fred Nalugoda, Stephen Watya, William Ddaaki, Alex Daama, Robert Bulamba, James Nkale, Emmanuel Kyasanku, Godfrey Kigozi, Grace Kigozi Nalwoga, Gertrude Nakigozi

**Affiliations:** 1 San Diego State University School of Public Health, San Diego, California, United States of America; 2 Africa Medical and Behavioral Sciences Organization, Kampala, Uganda; 3 Johns Hopkins Bloomberg School of Public Health, Baltimore, Maryland, United States of America; 4 Uganda Ministry of Health, Plot 6 Lourdel Road, Wandegeya, Kampala, Uganda; 5 Boston College School of Social Work, Chestnut Hill, Massachusettes, United States of America; 6 Africa Medical and Behavioral Sciences Organization, Nansana Hoima Road, Kampala, Uganda; 7 Uganda Ministry of Health, Plot 6 Lourdel Road, Wandegeya, Kampala, Uganda; 8 San Diego State University School of Public Health, San Diego, California, United States of America; 9 University of Zambia Great East Road Campus, Zambia; 10 Africa Medical and Behavioral Sciences Organization, Nansana Hoima Road, Kampala, Uganda; London School of Hygiene and Tropical Medicine Faculty of Epidemiology and Population Health, UNITED KINGDOM OF GREAT BRITAIN AND NORTHERN IRELAND

## Abstract

Early and ongoing stakeholder engagement is critical to the development and implementation of scalable behavioral health interventions. Our overarching goal in this implementation research study was to understand and document the process of planning and rolling out Common Elements Treatment Approach (CETA), a transdiagnostic mental health intervention, in Uganda. Guided by the Consolidated Framework for Implementation Research (CFIR) 2.0, we conducted in-depth interviews with multilevel stakeholders (healthcare providers, MOH members, clinic managers, implementing partners, *n = 20*) to understand and document the process of planning and rolling out CETA in Western Uganda. All interviews were audio recorded, transcribed, and analyzed thematically in Dedoose using a codebook based on CFIR 2.0 domains and constructs. Multilevel stakeholder engagement supported strategic decision making at each stage. Early endorsement of CETA by Uganda’s MOH was instrumental in facilitating program integration. Ministry of Health leadership guided facility selection, while clinic leaders identified trainees positioned to implement CETA. Training incorporated both didactic and participatory methods, including real-time adaptation of intervention content for contextual relevance and identification of “champion” trainees to serve as supervisors as part of the ongoing support supervision infrastructure. During rollout, an incremental implementation strategy facilitated integration into clinic workflows. Ongoing multilevel stakeholder supervision and technical assistance helped CETA deliverers troubleshoot challenges, maintain fidelity, and gain confidence. Together, these processes enabled expansion beyond the initial pilot sites, providing early evidence of stakeholder-perceived feasibility and identifying foundational conditions for scalability. Continued engagement with policymakers, those delivering CETA, and community stakeholders will be essential to refine CETA implementation and support integration into national mental health strategies. By reframing stakeholder engagement as an implementation strategy that builds enduring, locally led infrastructures, this study offers a replicable model for preparing behavioral health interventions for scale-up in low resource settings and advances implementation research in global mental health.

## Introduction

Mental and substance use disorders are leading causes of disability and mortality globally, with low and middle-income countries disproportionately burdened [1]. This burden is compounded by the fact that behavioral health is critically underfunded in precisely the settings and communities where need is greatest. In Uganda, only 5.1% of the national healthcare budget is allocated to mental health services [2], and there are on average two mental health providers per 100,000 residents, which is below the WHO recommendation of six per 100,000 [3,4]. Barriers such as the high cost of care, limited services, stigma and cultural differences in communicating sensitive issues (which can lead to underreporting and poor detection of symptoms with Western‑derived screening and diagnostic tools) further hinder mental health service use [5]. In high HIV prevalence settings, such as Uganda, the consequences of untreated mental illness and hazardous alcohol use also pose significant challenges to optimal antiretroviral therapy (ART) adherence. Moreover, they are also associated with poorer health outcomes, economic instability, and weakening social support systems [6–9]. Ongoing shifts in the global health funding landscape are placing additional strain on national systems, as governments are increasingly expected to fill funding gaps while balancing competing priorities. Collectively, these challenges emphasize the need for scalable, evidence-based programming that is cost-effective. Such programming must also provide training and supervision to enable lay providers to support integration into existing health infrastructure without placing additional strain on it.

These systemic challenges are compounded by historical and cultural factors that continue to shape mental health care in Uganda [10,11]. Combined with the limited number of psychiatric specialists in the country, this model created a care vacuum during the decolonization and deinstitutionalization era, resulting in an understaffed and under-resourced mental health system. Exacerbating these challenges, post-independence health system development occurred alongside periods of political instability and armed conflict, particularly in northern Uganda, contributing to a high burden of trauma-related mental health conditions [12,13]. Further mental health stigma often limits support-seeking from social networks, while persistent beliefs in supernatural causes of mental distress lead individuals to pursue non-medical solutions before seeking formal care [14]. These local explanatory models for how and why one experiences mental distress are often expressed through culturally specific idioms (e.g., “too many thoughts” as a way to describe worry) and behavioral manifestations, which may not align with Western diagnostic categories and can affect recognition, help-seeking, and engagement with formal mental health services [15,16]. Structural and gendered inequalities further shape vulnerability to mental health conditions and help seeking, underscoring the need for interventions that are also responsive to these social dynamics [17]. Recognizing the layered web of contextual dynamics is critical when introducing structured psychological interventions such as CETA [10].

The Common Elements Treatment Approach (CETA) is an evidence-based intervention specifically developed for use in low-resource settings [18]. Its modular design allows flexibility in both content and delivery, enabling components to be tailored to individual or group needs, symptom severity, and developmental stage [19]. This adaptability extends to its delivery model, which accommodates both professional and non-professional providers. Structured training and supervision protocols support fidelity while allowing for local adaptation and cultural relevance [20]. It can also be delivered in-person or via mobile platforms, increasing accessibility across diverse settings [21]. A core feature of the intervention is its apprenticeship-based training and supervision model, in which individuals receive intensive training (often 10 days) followed by weekly in-person and remote supervision. This model promotes ongoing skill development and sustained program fidelity. CETA further promotes scalability through both task-shifting (to lay counselors), and a train-the-trainer approach which supports in-country capacity building and long term sustainability [7,22]. Across multiple randomized trials, CETA has been shown to be acceptable to both those delivering the intervention and clients, adaptable to diverse delivery contexts, and effective in treating a range of mental health and substance use conditions.

Early and ongoing stakeholder engagement is critical to the development and implementation of scalable behavioral health interventions. Meaningful input from a diverse group of stakeholders, including clients, service deliverers, local officials, and health agency staff, at multiple stages of the process increases the relevance, feasibility and acceptability of evidence-based practices [23]. In the context of behavioral health, multilevel stakeholder engagement plays a particularly important role by improving research applicability and increasing the likelihood of policy uptake [24]. Stakeholder engagement is increasingly recognized as a core competency for implementation teams and is essential for ensuring ethical integrity, cultural responsiveness, overcoming implementation challenges, and sustainability [25]. Reflecting this, stakeholder engagement is included as a determinant, process, or construct in many widely used implementation frameworks (e.g., Exploration, Preparation, Implementation, and Sustainment (EPIS) [26], the Consolidated Framework for Implementation Research (CFIR) [27], and the integrated-Promoting Action on Research Implementation in Health Services (i-PARIHS) [28]). Despite this broad recognition of its importance, relatively few studies have documented stakeholder engagement processes in global mental health implementation, limiting opportunities for shared learning and evidence-informed practice. Importantly, there is also limited understanding of how these processes can extend beyond shaping immediate implementation outcomes and instead be leveraged to strengthening resilient, locally owned infrastructures that are critical for scalability and long-term sustainment in resource-constrained settings. This study responds to that gap by conceptualizing stakeholder engagement not only as a determinant of implementation success but also as an innovative strategy that builds infrastructure, fosters local ownership, and lays the groundwork for sustainable scale up in global mental health.

Our overarching goal in this implementation research study was to understand and document the process of planning and rolling out CETA in Uganda, with the aim of informing implementation in similar contexts and supporting scale up within Uganda. We situate this study within the broader need to build resilient, locally owned infrastructures for implementation. In doing so, we highlight stakeholder engagement as not only an implementation determinant but also as an implementation strategy that can strengthen long-term system capacity (e.g., supervision structures, decision-making processes, and feedback mechanisms). This article describes the pre-implementation and planning processes that guided CETA’s rollout for successful program delivery. A recent companion article reported on stakeholder perceptions of CETA’s long-term sustainability and impact in Uganda [29]. The CFIR 2.0 [30] provided the overarching framework for systematic analysis of determinants of pre-implementation and implementation success in this context. Findings can inform efforts to train future CETA deliverers, engage stakeholders, and adapt evidence-based interventions for similar settings. These insights may also serve as a blueprint for the broader rollout and scale up of CETA in Uganda and other low-resource settings.

## Methods

### Overview of CETA roll out

The CETA intervention has been described in detail previously [18]. Briefly, CETA uses a two-tiered design with individuals with mild to moderate symptoms first participating in a low threshold group intervention and those with more severe and persistent symptoms receiving subsequent ongoing individual sessions. In Uganda, the CETA pilot occurred between April 2022 and March 2023 across seven facilities in the Fort Portal region of Kabarole district, located in Western Uganda.

Preliminary stakeholder planning and engagement precedes CETA roll out in any new setting. First, policy level leaders were engaged to assess interest in CETA and establish a suitable implementation setting. The Fort Portal region, which is located in Western Uganda near the border with Democratic Republic of Congo, was selected given the presence of a regional referral hospital staffed with a psychiatric provider, which offered a critical referral pathway for CETA patients requiring specialized psychiatric care, and due to relatively high rates of nonadherence to HIV care among patients. Despite this resource, no evidence-based mental health treatments were available within the surrounding district-level health facilities. Facilities were also selected based on characteristics such as interest, capacity to deliver, and patient population needs. Leadership at these facilities was engaged, and support was provided to identify staff for CETA training. Of the trainees that participated in the initial two-week CETA training (n = 20), a subset was trained further to serve as site supervisors (n = 4). It was the responsibility of these supervisors to meet routinely with CETA deliverers and relay any challenges and successes back to key stakeholders providing ongoing technical assistance. In Uganda, this included the implementing partner organization, Johns Hopkins Program for International Education in Gynecology and Obstetrics (JHPIEGO), which facilitated the training as well as the CETA trainers, who traveled to Uganda for the in-person training.

### Study design

We employed an interpretivist paradigm and a constructivist epistemology. These design attributes were selected because the interpretivist paradigm aligns with our aim of understanding how stakeholders perceived and experienced implementation processes within context, while the constructivist epistemology emphasizes that reality is co-constructed through the perspectives of multiple stakeholders. This orientation is particularly appropriate for documenting early-stage implementation processes, where stakeholder perspectives are critical for informing adaptation and guiding future scale-up.

### Data collection

Methods for the present study have been described in detail previously in a companion manuscript [29]. In brief, between June 27, 2024, and September 24, 2024, we conducted in-depth interviews (IDIs) with key stakeholders that were involved in the planning and rollout of CETA in Uganda (n = 20). We determined that 20 interviews were sufficient to achieve both saturation and salience, based on prior work [31,32]. Participants represented multiple levels of engagement, including CETA-trained healthcare providers who delivered the intervention (hereafter “CETA deliverers”, n = 11), clinic leaders at implementation sites (n = 5), Ministry of Health (MOH) officials involved in the rollout (n = 2), and CETA developers and trainers (n = 2) who offered ongoing technical support throughout the pilot [33]). The social-behavioral research team at the Africa Medical and Behavioral Sciences Organization (AMBSO) led data collection. To support community engagement, AMBSO staff visited the district office in Kabarole District, met with local district stakeholders and secured approval to contact key individuals involved in CETA delivery and decision-making. A list of potential participants was generated with the support of contacts at CETA as well as clinic leadership. Participants were selected for their involvement in CETA and purposively sampled to ensure diverse stakeholder perspectives were captured. Interviews for most participants were conducted in person (18/20), except for the CETA developers/trainers (2/20), which were conducted over Zoom since these individuals reside outside of Uganda. Interviewers were male and female college graduates with training in qualitative research methods and prior experience conducting qualitative work in this setting and around public health specifically. All interviewers were native Ugandans, which facilitated cultural and linguistic competence and supported rapport-building with participants. Their familiarity with local health systems and community dynamics facilitated conversations with greater depth while minimizing the risk of misinterpretation. Informed consent was obtained prior to data collection and participants were compensated with 20,000 Uganda shillings (UGX) [~$6USD] for their time. Interviews were conducted in English and recorded via digital audio recorder.

Interview guides were semi-structured with question development guided by the Consolidated Framework for Implementation Research (CFIR) version 2.0 [30], an implementation determinants framework developed to conceptualize key constructs of implementation research [34]. CFIR 2.0 supports systematic assessment of implementation processes by organizing key contextual factors that influence implementation success into five domains (inner setting, outer setting, characteristics of individuals, innovation characteristics and implementation processes) comprised of 39 constructs. The CFIR was developed as a tool to advance implementation research through improved standardization of implementation constructs and promotion of an organizational framework and systematic analysis. CFIR can be applied before, during, and after implementation, and has been successfully used in a wide range of settings, including low-resource environments [35–37]. In the present study, the CFIR 2.0 was applied pre-implementation to understand barriers and facilitators to successful training and implementation at the inner and outer setting, process, and innovation domains. To support policy decisions and scale up, we also included questions to understand the CETA programs sustainability and recipient impact.

### Data analysis

Our analytic focus in the present study was to identify implementation strategies and contextual factors that facilitated roll-out of CETA and identify modifiable barriers that might be addressed in future iterations of CETA in this and similar settings. We transcribed and imported transcripts into Dedoose for analysis. We then developed a codebook *a priori* based on the CFIR 2.0. Two qualitative researchers (AM, DT) with experience in applying the CFIR 2.0 to qualitative data, coded the initial batch of 10% of transcripts (n = 2). Once we had assessed coding consistency using a consensus-based approach, clarified any ambiguous code definitions (to ensure consistent code application), and refined the codebook, AM independently coded the remaining transcripts. We utilized thematic analysis. CFIR constructs informed not only initial coding but also interpretation, with themes developed through synthesis across constructs and domains to identify cross-cutting implementation processes. Emergent themes were captured through memoing, and these memos were then discussed and interpreted with the larger research team.

Anticipated sample size was determined *a priori*. Saturation was operationalized as saturation in salience, a form of meaning saturation that prioritizes themes based on prominence across participants, defined as the point at which no additional highly relevant themes emerged across successive transcripts [38]. Salience was assessed iteratively through transcript review, coding, memoing and team discussion; as no new salient themes were identified during analysis of the full data corpus, additional data collection was not required.

To fully capture the depth of the findings, we divided the results into two companion papers. The first paper focused on the pilot implementation itself and perceived adaptations needed to optimize delivery and sustainability [29]. For the present paper, the unit of analysis was stakeholders’ accounts of pre-implementation planning and early rollout, including how they experienced, perceived, and participated in CETA implementation in Uganda. While the broader focus was CETA rollout, this analysis specifically examined stakeholder engagement, apprenticeship-based training and supervision, and incremental rollout as implementation processes supporting early delivery. At this stage, implementation success referred to stakeholder-perceived readiness for delivery, integration into clinic workflows, establishment of supervision and feedback routines, and acceptability of CETA training and support systems. Together, these papers offer a comprehensive account of the pre, during and post implementation process, from initial stakeholder engagement through the phaseout of technical assistance.

Deliberate steps were taken to support the trustworthiness of our findings, as defined by Lincoln and Guba [39]. Confirmability was enhanced through team-based coding, consensus-building meetings around emergent themes, and the use of illustrative participant quotations. Our collaborative analytic process, combined with an audit trail documenting key decisions, enhanced dependability. We employed purposive sampling and provided thick description of the study population and implementation context to support transferability. The analytic team was primarily composed of, and co-led by, Ugandan researchers with extensive contextual knowledge of Uganda’s health system. To minimize social desirability bias and power differentials, interviews were conducted by research staff not involved in program implementation, and participants were assured of confidentiality and independence from evaluation activities. The team engaged in ongoing reflexive discussions to consider how these roles and relationships may have influenced data interpretation. This ongoing reflexivity at both the individual and team levels, which contributed to the credibility and overall trustworthiness of our findings.

### Ethics approval and consent to participate

All study activities were approved by Clarke International University (CIUREC/059), Uganda National Council for Science and Technology (SS 4468) and San Diego State University (HS-2024–0088). All participants provided written informed consent prior to commencement of study activities. Additional information regarding the ethical, cultural, and scientific considerations specific to inclusivity in global research is included in the Supporting Information (S1 Checklist).

## Results

The majority of the twenty stakeholder participants were CETA deliverers (including counselors, a social worker, nurses, a clinician and occupational therapists), making up 55% of the sample (n = 11). Clinic leadership at CETA facilities comprised 25% (n = 5), while CETA developers and trainers (n = 2), along with MOH stakeholders (n = 2), each represented 10%. The average age of participants was 39.7 years (SD = 8.6). In terms of gender distribution, 65% of stakeholders identified as female and 35% as male. Given the small number of participants in the MOH and CETA developer/trainer strata, we collapsed these groups when attributing quotes to protect participant anonymity.

To enhance transparency in our CFIR 2.0 guided analysis, Table 1 provides a strategic illustration of how select key emergent themes were mapped to the framework’s domains and constructs during thematic synthesis.

**Table 1:** Strategic mapping of key emergent themes to CFIR 2.0 domains and constructs.

Key emergent theme	CFIR 2.0 Domain(s)	CFIR 2.0 Construct(s)	Implementation insight
Stakeholder engagement for strategic decision making	Process; Outer Setting	Engaging; Planning; Local Attitudes	Ministry of Health, district leadership, and facility stakeholders shaped site selection, trainee identification, and alignment with health system priorities.
Training adaptation and workforce preparation	Innovation; Individuals; Inner Setting	Adaptability; Innovation Deliverer; Access to Knowledge and Information; Innovation Complexity	Activities such as participatory role-play, tablet-based learning, and culturally responsive adaptations strengthened provider confidence, competence, and preparedness to deliver CETA.
Development of supervision and feedback infrastructure	Inner Setting; Process	Access to Knowledge and Information; Reflecting and Evaluating	Supervisor roles, technical assistance, and iterative feedback loops supported fidelity monitoring, real time troubleshooting, and implementation quality.
Incremental rollout within clinic workflows	Process	Doing	Stepwise implementation enabled integration into existing service delivery systems while allowing teams to identify and address operational barriers during early delivery.
Financing and resource burden	Outer Setting	Financing	Costs related to training, supervision, technology, and coordination were recognized as important considerations for sustainment and future scale-up.

Data were initially analyzed by CFIR construct and domain. Themes were then developed through cross-construct synthesis, allowing identification of patterns that spanned CFIR domains and reflected broader implementation processes. To enhance clarity and support the practical application of findings, the results are organized thematically and presented in the temporal order in which training and planning activities occurred. This structure reflects the implementation process and highlights how specific strategies and contextual factors evolved over time.

### Initial planning

Findings from the local attitudes (outer setting domain), planning and engaging (process domain) constructs emphasized the critical role played by key MOH stakeholders in facilitating the successful rollout of CETA from inception to implementation. Their contributions included selecting CETA as a promising evidence-based intervention for piloting, facilitating essential introductions to support planning and engagement efforts, assisting with training activities, and providing ongoing supervision support throughout the program’s rollout.

*I am not too sure all the actions [MOH supported] behind the scenes but I think we largely needed them to give us the go ahead to work within the HIV clinics so the actions they took I think they were approving the project with them and […] they connected us to site leaders I think also when we needed to sign some forms you know keeping sites engaged according to the numbers we needed to serve, the MOH stepped in and helped support there. I think largely in the initial stages of approving CETA as a chosen intervention and then approving and connecting us with the partner clinics. -Male, Trainer/MOH official category*

*I mean we gave [the CETA program] our staff, the health care workers, we gave them time to go and train which was quite long like weekly and we were also part of the supervision, we were part of the initial dissemination because we needed to share with the AIDS control program. How far we had gone with improving care for people living with HIV/AIDS as far as mental health is concerned, so we were with them to the different people who needed to hear and probably get their buy-in as well. - Female, Trainer/MOH official category*

### Stakeholder engagement to drive strategic decision making

Stakeholder engagement was characterized by strong leadership support, a top-down process for selecting pilot facilities, and active facility-level input in choosing CETA trainees, in coordination with the CETA training team. MOH leadership engagement was critical to program rollout and identification of facilities while facility-level leadership shaped the selection of trainees. Findings from the mid-level leaders (individuals’ domain) planning and engaging (process domain) constructs emphasized that once CETA was selected as the pilot innovation, key leadership from the MOH, Johns Hopkins University (JHU), and the implementing partner organization (JHPIEGO) collaborated to determine suitable pilot locations. As described by MOH senior leadership:

*We identified an area and a region where we would go. We chose the Rwenzori region because we had other work that we were doing as a department, so it was going to be easy for us to supervise […] but also, they had done well as far as mental health services are concerned, the regional referral was being managed by a psychiatrist. So, we said [Oh] that will be an easy entry point into the region...-Female, Trainer/MOH official category*

A top-down approach was used to identify participating facilities and eligible participants.

Senior level stakeholders at Hopkins, Jhpiego and the MOH engaged in discussion to determine where to implement the program and the initial few facilities to include in that region.

*Yeah, the [CETA] project was focusing on populations living with HIV and seeing that the mental health component of CETA would improve ART adherence and engagement with HIV treatment and so the MOH and JHPIEGO targeted Fort Portal regional hospital. One, because they expressed interest in getting more mental and behavioral support and two, they were having high rate of late HIV treatment among the population and so we decided that that was the place where we could make the most impact on the HIV outcomes with those findings. -Male, Trainer/MOH official category*

When asked about the key stakeholders at the facility level who were involved in championing the rollout of the CETA program, one MOH stakeholder emphasized the collective effort required for successful implementation:

*At the facility level, we had to talk with the head of the hospital, the MS (Medical superintendent […] I mean the hospital director was very key in there together with the people within the mental health unit within the regional referral hospital. -Female, Trainer/MOH official category*

The selection process for who would be engaged in CETA training was strategically guided by healthcare facility leadership to align with programmatic priorities and stakeholder objectives for program rollout. Individuals were carefully chosen to ensure representation from all potential facility entry points where individuals experiencing mental distress might seek care.

*They [JHPIEGO] were mostly interested in people [health workers] working with HIV clinics, mental health clinic, outpatient department and on wards because in outpatient department it is the starting point for the patients who come from out of the facility. So, when those who are very desperate come, they can first be handled from there. Ward personnels were chosen because they deal with inpatients associated with suicidal tendencies. Mental health unit [where I belong] was also included because it receives depressed clients and sometimes those with suicidal tendencies. -Female, CETA deliverer*

This multilevel engagement demonstrated how stakeholder involvement can extend beyond shaping immediate activities to building ownership and capacity within the health system by selecting facilities and individuals who had both the capacity and the commitment to implement CETA. It also facilitated the development of decision-making pathways and governance structures, including coordinated roles across MOH, implementing partners, and facility leadership, which supported site selection, resource allocation, and early implementation planning.

### Training process

Several factors supported the training process. Participants were motivated to learn CETA and recognized its value; training incorporated both didactic and applied techniques (e.g., clinical role-plays) alongside tablet-based tools; real-time adaptations were made to enhance cultural compatibility; and “champion participants” were identified to support staffing of supervisory roles. CETA deliverers were introduced to CETA right before training and implementation. Training occurred in most facilities during the initial phase in 2022, but the program was expanded to additional facilities in 2023 following feedback from CETA deliverers and other stakeholders, and improved patient outcomes. Findings under the engaging (implementation process domain), innovation deliverer (Individuals domain) and relative advantage (innovation domain) constructs suggested that once introduced to CETA, those invited to be trained in CETA were motivated to learn about the program (first quote) and saw a need for it at their facility (second quote).

*Since I was a counselor and social worker working with various people having different problems, Gender Based Violence and all other sorts of stress, I had a lot of interest to learn more [about CETA]. -Female, CETA deliverer*

*I embraced it as a very new approach since we had existing gaps in supporting people with HIV to adhere and overcome their mental health challenges. […] These patients lose hope and lose interest in taking these medications as well which at the end of the day impact their quality of life and so forth. -Male, CETA deliverer*

The initial training period lasted for two weeks and was conducted in-person at an offsite location by CETA trainers who traveled to Uganda specifically for the training exercises. Participant feedback indicated that the training format was well-received and effectively provided the necessary content to successfully deliver CETA, aligning with the access to knowledge and information constructs within the Inner Setting domain.

A key strength of the training was the use of tablets for content delivery, which was viewed as beneficial by both trainers (first quote) and trainees (second quote) alike, because they enhanced accessibility and engagement.

*The benefits from tech-based trainings are that it reduces burden on trainers and two, the trainees can like I said, they can move at their own pace and rewind […] -Male, Trainer/MOH official category*

*This [training] was good because it was short time and the tablets that we used had all the contents from that booklet I told you about, its contents were transferred into a software and it had some exercises which we could answer, there were also some modules where somebody testifies and we could answer those questions.-Female, CETA deliverer*

Participants described a combination of didactic and applied training techniques.

*During the training, we would first study and even write on the board, then they explain to us, then individuals would be chosen to go and participate to see if we had understood very well. -Female, CETA deliverer*

According to one trainer, the applied aspect of the training provided a valuable opportunity to identify elements of CETA that required tailoring to ensure compatibility with the Ugandan context. Both the symptom screening form and module content were adjusted in real time, incorporating feedback from those being trained to deliver CETA. This iterative process aligns with the Compatibility construct within the Inner Setting domain, facilitating a more contextually appropriate implementation.

*So CETA has the assessment of clients monitoring form which is a multi-problem screener and symptom monitoring tool. The Ugandan team did a pilot with some patients that ended with a quick adaptation of the assessment by changing the wording of some of the items to make sure that they are more contextually relevant and make sense to people. -Male, Trainer/MOH official category*

Training also offered opportunities to identify trainees who could be trained up as supervisors who would serve an essential role in supporting other CETA deliverers and liaising with technical assistance from the implementing partner organization and trainers during program roll out.

*We would try and identify what we would call champion participants, and these champion participants who seem to be getting the concepts very quickly, seem to be leaders, seem to be people that are quite knowledgeable/ conversant with CETA and beyond would then identify these people to be possibly be selected to be chosen as supervisors and then these supervisors would be given some kind of extra of supervision training […] on what one needs to be a supervisor, how to manage a group, how to provide clinical support. So that would be the general breakdown of how trainings happen. - Male, Trainer/MOH official category*

Some trainees initially felt overwhelmed by the complexity of CETA when it was introduced during training (innovation complexity and engaging constructs, innovation and implementation process domains, respectively). However, as one participant shared, despite the initial volume of information, the benefits of the program became clear. Several CETA deliverers even described how the CETA skills they gained through the training and ongoing implementation support helped them manage their own day-to-day challenges.

*My initial reaction while in the workshop, at first, we were* not *understanding things but later we picked up and we saw that it was interesting. Even us who attended the training, it has helped us a lot because we use those skills to manage our daily stress, you know the stresses are very many like on a daily basis or monthly. -Female, CETA deliverer*

Given the complexity of the CETA approach, which requires those delivering the intervention to learn several different modules, each with a multistep treatment process, a more extensive initial training is required. It was noted by stakeholders facilitating the training that extending the training to three weeks in future delivery could provide a less stressful learning environment for trainees, allowing for better absorption of the material [innovation complexity construct, innovation characteristics domain].

*In an ideal world I mean providing either a longer training [that] gives people more time to really digest the content would be my goal, be it a three weeks training then be gradual by size but we found that we were working with tight deadlines so we tried to have the training be done in two weeks which still may sometimes have some people feel a little bit behind but where that is compensated is in the practice right after the training people still get time to expose themselves with the content and the concepts and practice so then the apprenticeship model ties in really well to that.- Male, CETA Trainer/MOH official category*

This two-week training was jointly externally funded by JHU, JHPIEGO, and the MOH. While no direct costs were reported for the intervention itself, the costs associated with training activities and the ongoing supervision components throughout implementation were reported to be substantial, aligning with the financing construct within the Outer Setting domain. Stakeholders identified several resource demands associated with implementation, including in-person training logistics (e.g., travel and lodging), technology-supported learning (e.g., tablets and digital support), and ongoing supervision and coordination (e.g., airtime for communication). These operational demands were viewed as important considerations for broader rollout.

*The costs were quite high, and the money resources were inadequate we had to call people from the regions and then also because we were learning on tablets for notes and videos and some of our participants were not so familiar with the technology -Female, MOH Trainer/MOH official category*

Collectively, these processes contributed to the development of implementation infrastructure, including the identification and training of site-level supervisors, the establishment of structured supervision and mentorship roles, and the creation of locally adapted training materials to support ongoing delivery.

### Program implementation

Three program implementation subthemes emerged: incremental rollout facilitated integration into clinic workflows; ongoing supervision and technical assistance supported delivery quality and fidelity; and expansion to additional districts demonstrated feasibility and readiness for future scale-up. The roll-out of the CETA program was approached incrementally, starting with the identification of clinics with both capacities to support CETA and with the most need, followed by a strategic, step-by-step expansion [doing construct, implementation process domain]. As one individual involved at the leadership level of CETA explained, beginning with three clinics first, ensured the integration of CETA into the existing workflow while prioritizing the most urgent needs.

*So, it was about some strategic rollout in a way that captures not only those with the most needs but also fall into the existing workflow of the systems. We then… we always got feedback from the users and [CETA deliverer] to sum up the feedback cycles we were using […] So we maintained constant feedback cycles from the [CETA deliverer] and patients and stakeholders in order to continue to rollout CETA in a way that hopefully sets it up for sustainability. - Male, CETA Trainer/MOH official category*

Ongoing technical assistance was essential to support both CETA deliverers and broader program implementation. This model of sustained applied supervision differs from most traditional training programs and can be challenging to implement, mostly due to its novelty.

*I think what people found complicated about it was that it is not just a one off training and there is continuous supervision, I think most people even in the MOH are used to the trainings where people come in and they train a group of people on vaccines or something and then the trainers leave and the people come back ready to start using the program. With CETA it is a little different in that supervision […] is really the only way to build capacity in [CETA deliverer] especially when they are treating more severe mental health problems. -Male, Trainer/MOH official category*

Stakeholders highlighted that technical assistance was routinely provided through multiple channels: internally, by trained CETA supervisors within facilities who offered real-time support and troubleshooting, and externally, through continuous contact with CETA trainers (access to knowledge and information constructs within the Inner Setting domain).

*When we came from the training, we gathered as a team, we started revising bit by bit in order to understand how to manage the patients, we would sit like in a meeting, create scenarios of patient conditions then we make consultations, so it was a bit complicated but as time went by we learnt how to handle. -Female, CETA deliverer*

In some instances, supervisors were even brought in to support implementation at the delivery level.

*During the introduction part of the session, as a [CETA deliverer], you would inform the client about the supervisor as someone as a reference in case of any challenging situation. This could help to build confidence in a client that the supervisor is also part of the team. So, in case of any challenging situation with the client, we [CETA deliverer] could make a phone call to consult from the supervisor. -Female, CETA deliverer*

Role-playing activities significantly improved CETA deliverer’s confidence in carrying out the intervention steps. The Trauma module was described as challenging to implement due to cultural norms that discourage open discussion of difficult and sensitive topics, such as rape, highlighting how social and historical dynamics influence intervention delivery and the need to adapt the module flexibly to better align with the local context.

*After the training, we formed groups and went through the steps thoroughly and after everybody would present to make sure, we don’t forget [anything] when it comes to attending to the clients. We went through the steps for more than two weeks because after the training, some people were half baked [meaning they didn’t get everything] so after the training we had to come again and repeat them with our supervisors. The supervisors would demonstrate as we follow and after everybody would present. During the presentation, the supervisor would act as a client whereas a trainee as a counselor and then change respectively. This helped us to be more conversant with the steps before facing the clients. -Female, CETA deliverer*

Nevertheless, many participants reported that once trained, the intervention was not difficult to implement, both in terms of content and delivery, with several noting that CETA streamlined their work processes. This ease of use was attributed to the clarity of the intervention manual and the availability of ongoing supervision and training support.

*Actually, this program did not have a lot of complexity, it actually enhanced our skills because for substance use according to the manual it remains as substance use among people living with HIV, among adolescents, gender-based violence issues then fear and anxiety, it really added us more skills as providers since it looked into the issues that were not being tackled. -Male, CETA deliverer*

*The good thing about CETA was that one did not require thinking about it through their head but it was rather about reading about the next steps based on the manual that for example if someone presented with suicidal ideations and at the same time had trauma, I would first consider suicidal ideations and not only that but also for anything that arose one could just make a call [for technical assistance].- Female, clinic leadership stakeholder*

Once CETA was successfully implemented in the initial clinics, a second round of training occurred to expand the program to the broader region, suggesting potential for replication and highlighting conditions that may support future scalability.

*We started out with Rwamwanja and Fort Portal and I felt clearly that the MOH and JHPIEGO felt that we could do more and so we scaled out to a few other districts […] I think we had about 64 clinics that we working with […] so it showed that it was effective in scaling up and we were not limited to only specialized providers but we could use people that may have a little less training as well. -Male, Trainer/MOH official category*

Collectively these strategies illustrate how pre-implementation planning contributed to the establishment of key infrastructure components (e.g., supervision systems, governance processes, and continuous feedback mechanisms) that, while tested at small scale, reflect foundational structures needed for sustained delivery.

## Discussion

Consistent with the broader literature on the mental health treatment gap in sub-Saharan Africa, access to mental health services remains severely constrained [40–43]. Critical perspectives highlight that interventions designed in high-income contexts often overlook local explanatory models, sociocultural norms, and structural inequalities; for example, Quarshie (2022) shows how Western psychiatric policies in West Africa were implemented without engaging local care practices [44], while Kohrt and Mendenhall (2016) emphasize the importance of integrating local concepts of distress and social context into global mental health interventions [45]. This study details a process of rigorous, early and ongoing stakeholder engagement functioning as an functioning as an implementation strategy that contributed to the development of key infrastructure components rather than only a contextual determinant of programmatic success. This engagement, when paired with structured planning and participatory training, created early conditions that support integration into public health systems and provide a foundation for sustainability. Prior work has emphasized how structured pre-implementation activities (e.g., training, stakeholder engagement, and coordinated planning) can provide a roadmap for successful rollout of complex interventions in public health systems [46,47]. By drawing on the Consolidated Framework for Implementation Research (CFIR 2.0) [30], our analysis offers actionable guidance that can inform the scale up of transdiagnostic interventions such as the Common Elements Treatment Approach (CETA) in other low-resource settings.

Early endorsement of CETA by Uganda’s MOH was instrumental in facilitating program integration and legitimacy. Prior work suggests that high-level leadership buy-in and community ownership are key determinants of successful program implementation in LMICs, especially where interventions must be embedded within a strained health system [48–50]. Early and ongoing MOH engagement can enhance sustainability, accelerate and improve implementation processes, increase the likelihood of scaling, and support the integration of interventions into existing health services. In the present study, MOH actors were instrumental in selecting pilot sites, mobilizing healthcare providers to participate, and ensuring alignment with national priorities such as HIV care and mental health integration. Such early, high-level engagement from policy stakeholders illustrates the critical influence of factors within the Outer Setting domain, which encompasses how external mandates, recommendations, and priorities shape early and pre-implementation processes. These findings are also consistent with other studies where early alignment with ministry agendas facilitated acceptability and built ownership at multiple levels, including the PRIME Mental Health Programme in Ethiopia, India, Nepal, South Africa, and Uganda [51], and Nigeria’s Comprehensive Community Mental Health Programme (CCMHP) [52].

CETA’s training model was widely perceived as effective and empowering, fostering both technical competence and confidence among those trained to deliver CETA through its skills-based and participatory format. Stakeholders emphasized that the hands-on, participatory nature of the training process played a key role in preparing trainees to deliver complex, modular care in diverse clinical settings. Beyond skill acquisition, the training also functioned as a space for collaborative adaptation, where content and tools were refined in real-time to better align with local contexts. This iterative, dynamic and feedback-driven approach not only strengthened cultural relevance but also deepened stakeholder investment by fostering a sense of co-ownership. It is also supported by implementation science, which increasingly emphasizes collaborative, context-sensitive adaptation as a key enabler of successful and sustainable intervention delivery research [53–55]. Furthermore, the CETA “apprenticeship” model, which leverages train-the-trainer and local supervisor models supported long-term sustainability by identifying and cultivating future supervisors, who could maintain program fidelity and offer technical support post-implementation [56]. These elements enhanced the intervention’s implementation readiness by strengthening inner setting constructs such as access to knowledge and information, compatibility with existing workflows, and individual confidence in intervention delivery. These features also underscore how thoughtfully designed training and ongoing support supervision feedback can serve as both a platform for capacity building and a foundation for durable, locally owned implementation.

Although CETA rollout in Uganda was a pilot, the deliberate, incremental, stepwise implementation approach, which prioritized facilities with both high need and implementation capacity, aligns with recommendations in the scale up literature [56–58] where pilot activities are designed to develop a “scalable unit”, that can facilitate smoother transition to broader implementation. During the CETA rollout, adaptation was both proactive (i.e., during pre-implementation training) and responsive (i.e., through ongoing technical assistance and meetings with supervisors) to challenges. Similar approaches have been recommended in other implementation science studies to reduce system disruption and maintain quality during scale up, particularly through real-time modifications and context-driven iteration during pilot phases [57]. A key pillar of this strategy was sustained technical assistance, delivered both internally through trained in-facility supervisors and externally via ongoing communication with CETA trainers. This dual-channel model of applied supervision represented a notable departure from conventional training programs, providing real-time guidance and reinforcing fidelity throughout early implementation. Role-playing exercises were particularly effective in building provider confidence, helping translate training into practice. Collectively, these elements created a supportive ecosystem that facilitated the smooth integration of a complex intervention into routine care [59].

Despite the overall success of the rollout, several challenges to long-term scalability remain. Stakeholders identified training logistics, technology needs, and ongoing supervision as important resource demands for broader implementation. Potential strategies to reduce these burdens include certifying local CETA trainers through the train-the-trainer model, embedding training within existing professional development systems, and reducing reliance on external technical assistance as local supervisory capacity strengthens. While this pilot produced individuals trained to deliver CETA and supervise CETA it did not establish in-country capacity to independently lead future trainings. Establishing trainers in Uganda will be the first critical step to a scalable CETA program. In addition, stakeholders underscored the value of embedding CETA training into existing national systems for professional development and supervision. Institutionalizing these efforts within Uganda’s healthcare workforce infrastructure would provide long-term legitimacy and budgetary sustainability. Finally, expanding the roll out in Uganda to include community health workers (locally known as Village Health Teams or Community Health Extension Workers (CHEWs) may offer a pathway to extend reach to rural or underserved areas, consistent with successful community-based mental health programs documented in other parts of sub-Saharan Africa.

### Strengths and limitations

This study has several strengths and limitations. While it captures a broad range of perspectives from stakeholders involved in training, engagement, and planning, it does not include feedback from CETA recipients, limiting our understanding of perceived benefits or challenges from the end-user perspective. This is something that should be captured as a part of post-implementation assessment in future work. Additionally, interviews were conducted retrospectively (i.e., after the pilot period) which may have introduced recall or social desirability biases; however, these risks were mitigated through independent interviewers not affiliated with any entity involved in CETA roll out, confidentiality assurances and neutral probing. While qualitative findings offer rich contextual insights, they are not intended to be generalizable across all Ugandan health settings. Nonetheless, the alignment of identified barriers and facilitators with broader literature on mental health implementation in LMICs strengthens the transferability of our findings.

## Conclusions

The urgent need for scalable, evidence-based mental health interventions in Uganda continues to grow, particularly in underserved communities burdened by high rates of hazardous alcohol use, depression, and limited access to behavioral health services. Looking ahead, continued engagement with policymakers, those delivering CETA, and community stakeholders will be essential to refine CETA’s implementation and ensure its integration into national mental health strategies. As Uganda and similar countries confront the dual challenges of high mental health burden and limited-service infrastructure, interventions like CETA offer a promising path forward, particularly when accompanied by robust planning, tailored training, and inclusive stakeholder engagement. By reframing stakeholder engagement as an implementation strategy that builds enduring and locally owned infrastructures offers a replicable model for scaling behavioral health interventions in low resource settings and advances implementation research in global mental health. Future research should explore how these implementation foundations translate into sustained service delivery, clinical impact, and system-wide change. Ultimately, building resilient, locally owned implementation infrastructures is critical for ensuring that CETA and similar programs reach the communities that need them most.

### Contributions to the Literature

- Demonstrates that stakeholder engagement is not only a construct but also a dynamic process that facilitates timely adaptation of implementation strategies and intervention content.
- Applies CFIR 2.0 to systematically present how stakeholder engagement supported planning and implementation, providing a blueprint for scaling CETA in Uganda and other low resource settings.
- Offers findings and recommendations that are particularly timely given global shifts in funding priorities that disproportionately affect low- and middle-income countries, along with calls to integrate service delivery and transition to locally driven programming where practical guidance on how to achieve this remains limited.

## Supporting information

S1 Checklist Inclusivity in global research. (DOCX)

## References

[1] Lu J, Xu X, Huang Y, Li T, Ma C, Xu G, et al. Prevalence of depressive disorders and treatment in China: a cross-sectional epidemiological study. Lancet Psychiatry. 2021;8(11):981–90. doi: 10.1016/S2215-0366(21)00251-0 34559991

[2] Ministry of Finance Planning and Economic Development. National budget framework paper FY 2020/21- FY 2024/25. Ministry of Finance Planning and Economic Development; 2020.

[3] WHO. Mental health atlas. Geneva: World Health Organization; 2021.

[4] World Health Organization. Mental Health Atlas 2017. 2018.

[5] Bolton P, Bass J, Neugebauer R, Verdeli H, Clougherty KF, Wickramaratne P, et al. Group interpersonal psychotherapy for depression in rural Uganda: a randomized controlled trial. JAMA. 2003;289(23):3117–24. doi: 10.1001/jama.289.23.3117 12813117

[6] World Health Organization. World mental health report: transforming mental health for all. Geneva: WHO; 2022.

[7] World Health Organization. Global status report on alcohol and health and treatment of substance use disorders. Geneva: World Health Organization; 2024.

[8] Velloza J, Kemp CG, Aunon FM, Ramaiya MK, Creegan E, Simoni JM. Alcohol use and antiretroviral therapy non-adherence among adults living with HIV/AIDS in Sub-Saharan Africa: a systematic review and meta-analysis. AIDS Behav. 2020;24(6):1727–42. doi: 10.1007/s10461-019-02716-0 31673913 PMC7190427

[9] Gonzalez JS, Batchelder AW, Psaros C, Safren SA. Depression and HIV/AIDS treatment nonadherence: a review and meta-analysis. J Acquir Immune Defic Syndr. 2011;58(2):181–7. doi: 10.1097/QAI.0b013e31822d490a 21857529 PMC3858003

[10] Antic A, Abarca-Brown G, Moghnieh L, Rajpal S. Toward a new relationship between history and global mental health. SSM - Mental Health. 2023;4.

[11] Pringle Y. Psychiatry and decolonisation in Uganda. London, UK: Palgrave MacMillan; 2019.

[12] Amone-P’olak K, Jones PB, Abbott R, Meiser-Stedman R, Ovuga E, Croudace TJ. Cohort profile: mental health following extreme trauma in a northern Ugandan cohort of War-Affected Youth Study (The WAYS study). Springerplus. 2013;2(1):300. doi: 10.1186/2193-1801-2-300 23888271 PMC3717156

[13] Luo J, Zamar DS, Ogwang MD, Muyinda H, Malamba SS, Katamba A, et al. Cango Lyec (Healing the Elephant): probable post-traumatic stress disorder (PTSD) and depression in Northern Uganda five years after a violent conflict. J Migr Health. 2022;6:100125. doi: 10.1016/j.jmh.2022.100125 35832466 PMC9272377

[14] Omona K, Bashabire NBM, Bulamu R, Mugabe B, Balamaga SS, Akurut BE. Mental health in low-income countries: a call to improve mental health in Uganda. PLOS Ment Health. 2025;2(4):e0000313. doi: 10.1371/journal.pmen.0000313 41661970 PMC12798614

[15] Mendenhall E, Rinehart R, Musyimi C, Bosire E, Ndetei D, Mutiso V. An ethnopsychology of idioms of distress in urban Kenya. Transcult Psychiatry. 2019;56(4):620–42. doi: 10.1177/1363461518824431 30672722

[16] Miller AP, Ziegel L, Mugamba S, Kyasanku E, Wagman JA, Nkwanzi-Lubega V, et al. Not enough money and too many thoughts: exploring perceptions of mental health in two ugandan districts through the mental health literacy framework. Qual Health Res. 2021;31(5):967–82. doi: 10.1177/1049732320986164 33451275 PMC8628861

[17] Morgan R, Ayiasi RM, Barman D, Buzuzi S, Ssemugabo C, Ezumah N, et al. Gendered health systems: evidence from low- and middle-income countries. Health Res Policy Syst. 2018;16(1):58. doi: 10.1186/s12961-018-0338-5 29980230 PMC6035473

[18] Murray LK, Dorsey S, Haroz E, Lee C, Alsiary MM, Haydary A. A common elements treatment approach for adult mental health problems in low- and middle-income countries. Cogn Behav Pract. 2014;21(2):111–23. 25620867 10.1016/j.cbpra.2013.06.005 PMC4304666

[19] Murray LK, Kane JC, Glass N, Skavenski van Wyk S, Melendez F, Paul R, et al. Effectiveness of the Common Elements Treatment Approach (CETA) in reducing intimate partner violence and hazardous alcohol use in Zambia (VATU): a randomized controlled trial. PLoS Med. 2020;17(4):e1003056. doi: 10.1371/journal.pmed.1003056 32302308 PMC7164585

[20] Kendall PC, Beidas RS. Smoothing the trail for dissemination of evidence-based practices for youth: flexibility within fidelity. Prof Psychol Res Pract. 2007;38(1):13–20. doi: 10.1037/0735-7028.38.1.13

[21] Munthali-Mulemba S, Figge CJ, Metz K, Kane JC, Skavenski S, Mwenge M, et al. Experiences and perceptions of telephone-delivery of the common elements treatment approach for mental health needs among young people in Zambia during the COVID-19 pandemic. Front Public Health. 2022;10:906509. doi: 10.3389/fpubh.2022.906509 36311612 PMC9610836

[22] World Health Organization. Task shifting to tackle health worker shortages. 2007.

[23] Green AE, Aarons GA. A comparison of policy and direct practice stakeholder perceptions of factors affecting evidence-based practice implementation using concept mapping. Implement Sci. 2011;6:104. doi: 10.1186/1748-5908-6-104 21899754 PMC3178500

[24] Pandi-Perumal SR, Zeller JL, Parthasarathy S, Edward Freeman R, Narasimhan M. Herding cats and other epic challenges: creating meaningful stakeholder engagement in community mental health research. Asian J Psychiatr. 2019;42:19–21. doi: 10.1016/j.ajp.2019.03.019 30939395

[25] Murphy J, Qureshi O, Endale T, Esponda GM, Pathare S, Eaton J, et al. Barriers and drivers to stakeholder engagement in global mental health projects. Int J Ment Health Syst. 2021;15(1):30. doi: 10.1186/s13033-021-00458-y 33812375 PMC8019163

[26] Aarons GA, Hurlburt M, Horwitz SM. Advancing a conceptual model of evidence-based practice implementation in public service sectors. Adm Policy Ment Health. 2011;38(1):4–23. doi: 10.1007/s10488-010-0327-7 21197565 PMC3025110

[27] Damschroder LJ, Aron DC, Keith RE, Kirsh SR, Alexander JA, Lowery JC. Fostering implementation of health services research findings into practice: a consolidated framework for advancing implementation science. Implement Sci. 2009;4:50. doi: 10.1186/1748-5908-4-50 19664226 PMC2736161

[28] Harvey G, Kitson A. PARIHS revisited: from heuristic to integrated framework for the successful implementation of knowledge into practice. Implement Sci. 2016;11:33. doi: 10.1186/s13012-016-0398-2 27013464 PMC4807546

[29] Miller AP, Nakigozi G, Menya E, Figge C, Lukwata H, Byansi W, et al. Implementation, sustainability, and impact of common elements treatment approach in Ugandan healthcare facilities. SSM - Mental Health. 2025;8:100569. doi: 10.1016/j.ssmmh.2025.100569 PMC13289709 42344436

[30] Damschroder LJ, Reardon CM, Widerquist MAO, Lowery J. The updated consolidated framework for implementation research based on user feedback. Implement Sci. 2022;17(1):75. doi: 10.1186/s13012-022-01245-0 36309746 PMC9617234

[31] Guest G, Bunce A, Johnson L. How many interviews are enough?: an experiment with data saturation and variability. Field Methods. 2006;18(1):59–82.

[32] Hennink MM, Kaiser BN, Marconi VC. Code saturation versus meaning saturation: how many interviews are enough?. Qual Health Res. 2017;27(4):591–608. 27670770 10.1177/1049732316665344 PMC9359070

[33] Murray LK, Dorsey S, Bolton P, Jordans MJ, Rahman A, Bass J, et al. Building capacity in mental health interventions in low resource countries: an apprenticeship model for training local providers. Int J Ment Health Syst. 2011;5(1):30. doi: 10.1186/1752-4458-5-30 22099582 PMC3284435

[34] Damschroder LJ, Aron DC, Keith RE, Kirsh SR, Alexander JA, Lowery JC. Fostering implementation of health services research findings into practice: a consolidated framework for advancing implementation science. Implement Sci. 2009;4:50. doi: 10.1186/1748-5908-4-50 19664226 PMC2736161

[35] English M, Nzinga J, Mbindyo P, Ayieko P, Irimu G, Mbaabu L. Explaining the effects of a multifaceted intervention to improve inpatient care in rural Kenyan hospitals--interpretation based on retrospective examination of data from participant observation, quantitative and qualitative studies. Implement Sci. 2011;6:124. doi: 10.1186/1748-5908-6-124 22132875 PMC3248845

[36] Adamu AA, Uthman OA, Gadanya MA, Wiysonge CS. Using the consolidated framework for implementation research (CFIR) to assess the implementation context of a quality improvement program to reduce missed opportunities for vaccination in Kano, Nigeria: a mixed methods study. Hum Vaccin Immunother. 2020;16(2):465–75. doi: 10.1080/21645515.2019.1654798 31424313 PMC7062445

[37] Means AR, Kemp CG, Gwayi-Chore M-C, Gimbel S, Soi C, Sherr K, et al. Evaluating and optimizing the consolidated framework for implementation research (CFIR) for use in low- and middle-income countries: a systematic review. Implement Sci. 2020;15(1):17. doi: 10.1186/s13012-020-0977-0 32164692 PMC7069199

[38] Hennink MM, Kaiser BN, Marconi VC. Code saturation versus meaning saturation: how many interviews are enough?. Qual Health Res. 2017;27(4):591–608. 27670770 10.1177/1049732316665344 PMC9359070

[39] Lincoln Y, Guba E. Naturalistic inquiry. Newbury Park, CA: Sage Publications; 1985.

[40] Williams SA, Baldeh M, Bah AJ, Dennis F, Robinson DR, Adeniyi YC. Pathways to mental health services across local health systems in sub-Saharan Africa: findings from a systematic review. PLoS One. 2025;20(6):e0324064. doi: 10.1371/journal.pone.0324064 40526705 PMC12173185

[41] Nicholas A, Joshua O, Elizabeth O. Accessing Mental Health Services in Africa: Current state, efforts, challenges and recommendation. Ann Med Surg (Lond). 2022;81:104421. doi: 10.1016/j.amsu.2022.104421 35996570 PMC9387063

[42] Rammouz I, Merzouki M, Najimi M, Doufik J, Sylla A, Fadel CM, et al. Revolutionising mental health in Africa: bridging gaps and breaking down barriers – CORRIGENDUM. BJPsych Int. 2025;:1–1. doi: 10.1192/bji.2025.1008841953520

[43] Komu CK, Ngigi M, Melson AJ. Barriers and facilitators to accessing mental health services for adults in sub‐saharan Africa: a systematic review. Mental Health Science. 2025;3(1). doi: 10.1002/mhs2.70006

[44] Quarshie NO. Psychiatry on a shoestring: West Africa and the global movements of deinstitutionalization. Bull Hist Med. 2022;96(2):237–65. doi: 10.1353/bhm.2022.0023 35912620

[45] Kohrt BA, Mendenhall E, Brown PJ. How anthropological theory and methods can advance global mental health. Lancet Psychiatry. 2016;3(5):396–8. doi: 10.1016/S2215-0366(16)00046-8 27155506 PMC4957809

[46] Goodrich D, Miake-Lye I, Braganza M. The QUERI Roadmap for Implementation and Quality Improvement. Washington (DC): Department of Veterans Affairs (US); 2020. 33400452

[47] Alley ZM, Chapman JE, Schaper H, Saldana L. The relative value of pre-implementation stages for successful implementation of evidence-informed programs. Implement Sci. 2023;18(1):30. doi: 10.1186/s13012-023-01285-0 37480144 PMC10362770

[48] Ojo T, Kabasele L, Boyd B, Enechukwu S, Ryan N, Gyamfi J, et al. The role of implementation science in advancing resource generation for health interventions in low- and middle-income countries. Health Serv Insights. 2021;14:1178632921999652. doi: 10.1177/1178632921999652 33795935 PMC7970459

[49] Giebel C, Gabbay M, Shrestha N, Saldarriaga G, Reilly S, White R, et al. Community-based mental health interventions in low- and middle-income countries: a qualitative study with international experts. Int J Equity Health. 2024;23(1):19. doi: 10.1186/s12939-024-02106-6 38308294 PMC10835969

[50] Nwaozuru U, Murphy P, Richard A, Obiezu-Umeh C, Shato T, Obionu I, et al. The sustainability of health interventions implemented in Africa: an updated systematic review on evidence and future research perspectives. Implement Sci Commun. 2025;6(1):39. doi: 10.1186/s43058-025-00716-x 40200368 PMC11980204

[51] Breuer E, De Silva MJ, Fekadu A, Luitel NP, Murhar V, Nakku J, et al. Using workshops to develop theories of change in five low and middle income countries: lessons from the programme for improving mental health care (PRIME). Int J Ment Health Syst. 2014;8:15. doi: 10.1186/1752-4458-8-15 24808923 PMC4012094

[52] Ryan GK, Nwefoh E, Aguocha C, Ode PO, Okpoju SO, Ocheche P, et al. Partnership for the implementation of mental health policy in Nigeria: a case study of the comprehensive community mental health programme in Benue state. Int J Ment Health Syst. 2020;14:10. doi: 10.1186/s13033-020-00344-z 32110245 PMC7033947

[53] Leeman J, Rohweder C, Lee M, Brenner A, Dwyer A, Ko LK, et al. Aligning implementation science with improvement practice: a call to action. Implement Sci Commun. 2021;2(1):99. doi: 10.1186/s43058-021-00201-1 34496978 PMC8424169

[54] McKay MM, Sensoy Bahar O, Ssewamala FM. Implementation science in global health settings: collaborating with governmental and community partners in Uganda. Psychiatry Res. 2020;283:112585. doi: 10.1016/j.psychres.2019.112585 31590906 PMC6954316

[55] Aarons GA, Green AE, Palinkas LA, Self-Brown S, Whitaker DJ, Lutzker JR, et al. Dynamic adaptation process to implement an evidence-based child maltreatment intervention. Implement Sci. 2012;7:32. doi: 10.1186/1748-5908-7-32 22512914 PMC3436717

[56] Murray LK, Dorsey S, Bolton P, Jordans MJ, Rahman A, Bass J, et al. Building capacity in mental health interventions in low resource countries: an apprenticeship model for training local providers. Int J Ment Health Syst. 2011;5(1):30. doi: 10.1186/1752-4458-5-30 22099582 PMC3284435

[57] Zamboni K, Schellenberg J, Hanson C, Betran AP, Dumont A. Assessing scalability of an intervention: why, how and who? Health Policy Plan. 2019;34(7):544–52. 31365066 10.1093/heapol/czz068 PMC6788216

[58] Leeman J, Draeger LB, Lyons K, Pham L, Samuel-Hodge C. Tailoring implementation strategies for scale-up: preparing to take the Med-South Lifestyle program to scale statewide. Front Health Serv. 2022;2:934479. doi: 10.3389/frhs.2022.934479 36925769 PMC10012719

[59] Ray ML, Wilson MM, Wandersman A, Meyers DC, Katz J. Using a training-of-trainers approach and proactive technical assistance to bring evidence based programs to scale: an operationalization of the interactive systems framework’s support system. Am J Community Psychol. 2012;50(3–4):415–27. doi: 10.1007/s10464-012-9526-6 22711269

